# Bacteriospermia and Its Impact on Basic Semen Parameters among Infertile Men

**DOI:** 10.1155/2016/2614692

**Published:** 2016-01-06

**Authors:** Sangeetha Vilvanathan, Balan Kandasamy, Abiramy Lakshmy Jayachandran, Sarasa Sathiyanarayanan, Vijayalakshmi Tanjore Singaravelu, Veeraraghavan Krishnamurthy, Vanithadevi Elangovan

**Affiliations:** ^1^Department of Microbiology, Karpaga Vinayaga Institute of Medical Sciences and Research Centre, Chinna Kolambakkam Post, Madurantakam Taluk, Kanchipuram, Tamil Nadu 603 308, India; ^2^Department of Medical Gastroenterology, Rajiv Gandhi Government General Hospital, Chennai, Tamil Nadu 600 003, India

## Abstract

*Introduction*. Semen analysis is considered as the surrogate marker for male fecundity while assessing infertile men. There are several reasons for altered semen quality and bacteriospermia could be one among them. Thereby the aim of our work is to study the semen culture and its impact on semen parameters among infertile men.* Materials and Methods*. Semen samples were collected from men attending infertility clinic. Semen parameters were analysed based on WHO guidelines. Also, samples were subjected to culture using standard bacteriological techniques.* Results*. A total of 85 samples were collected. A number of 47 (55.30%) had normal sperm count, 37 (43.50%) had oligozoospermia, and one (1.17%) had azoospermia. Teratozoospermia was the most common abnormality observed (81.17%) followed by asthenozoospermia (28.23%). The prevalence of bacteriospermia was 35.3%.* Enterococcus faecalis* (30%) was the most common organism isolated followed by Coagulase negative* Staphylococcus* (23.33%),* Staphylococcus aureus* (20%), and* E. coli* (10%). Other less frequently isolated organisms were* Klebsiella pneumoniae* (6.66%),* Proteus* sp. (6.66%), and* Citrobacter* sp. (3.33%).* Conclusion*. The presence of asymptomatic bacteriospermia did not correlate with abnormal semen parameters.

## 1. Introduction

Infertility, a condition being different from various existing health related issues, in involving psychological and social aspects, affects 10–15% of couples in the reproductive age group [1]. Both sexes are more or less equally involved. Men, either alone or along with their female partners, contribute to 40–45% cases of infertility [2]. Furthermore, infectious etiologies involving bacteria, virus, fungi, and protozoa contribute to 15% of male factor infertility [3–5].

Bacteriospermia affects the normal fertility process by any of these following mechanisms: deterioration of spermatogenesis, decreased sperm motility, altered acrosome reaction, altered morphology, formation of reactive oxygen species leading to increased DNA fragmentation index, formation of antisperm antibodies due to breach in the blood-testes barrier, and genital tract obstruction due to inflammation and fibrosis [5–8].

Majority of these genitourinary tract infections remain asymptomatic, thereby leading to a dilemma whether to treat these patients or not. Evidences are being accumulating on the association of asymptomatic bacteriospermia and altered semen quality. Therefore, in this study, we have planned to determine the seminal patterns and prevalence of bacteriospermia and their impact on semen quality among infertile men.

## 2. Materials and Methods

Semen samples from 85 male partners of infertile couple attending infertility clinic in a tertiary care hospital were collected during the study period of June 2014 to June 2015. Samples were collected in sterile containers by masturbation after a minimum abstinence period of 3 days. None of the patients had taken prior antibiotics.

Semen parameters such as appearance, volume, pH, viscosity, liquefaction, count, motility, morphology, presence of other cells like epithelial cell or round cell, and sperm agglutination were recorded according to the WHO guidelines [9]. Gram stain and culture of the samples in blood agar and MacConkey agar were done in microbiology laboratory within 3 hours of specimen collection as per WHO guidelines [10]. Cultures were incubated at 37°C. Those organisms which were isolated in a concentration of >10^3^ cfu/mL were considered as significant [11]. Antimicrobial sensitivity testing by Kirby-Bauer disc diffusion method was done to the isolated organisms as per standard guidelines [12].

## 3. Statistical Analysis

The prevalence of bacteriospermia was calculated from the proportion of positive cases to the total number of study populations and expressed as percentages. The relationship between bacteriospermia and other semen parameters was analysed using Chi-square test.

## 4. Results

During the study period, 85 semen samples were received in the infertility clinic from male partners of infertile couples. The mean age group of the study population was 33.09. A number of 18 (21.17%) men were in the age group of <30 years and 67 (78.82%) men were belonging to the age group of >30 years.

**Figure 1:**
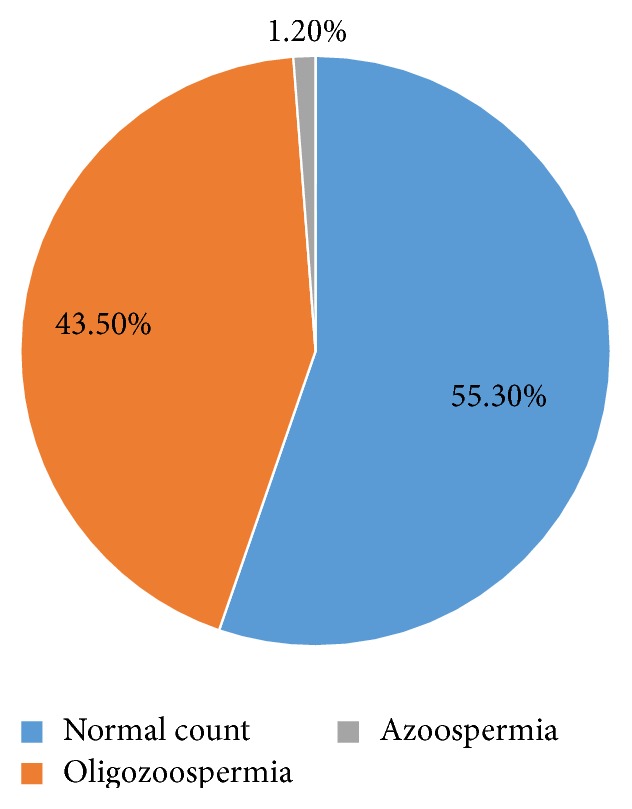
Semen density pattern of the study population.

Among the 85 specimens examined based on WHO guidelines [9], 47 (55.30%) had normal sperm count, 37 (43.50%) had oligozoospermia (spermatozoa concentration less than 15 million per milliliter), and 1 (1.17%) had azoospermia (absence of spermatozoa in the ejaculate) (Figure 1).

Morphological abnormality (teratozoospermia) was the most common abnormality observed in 69 (81.17%) subjects followed by abnormalities in the motility (asthenozoospermia) in 24 (28.23%) subjects.

**Figure 2:**
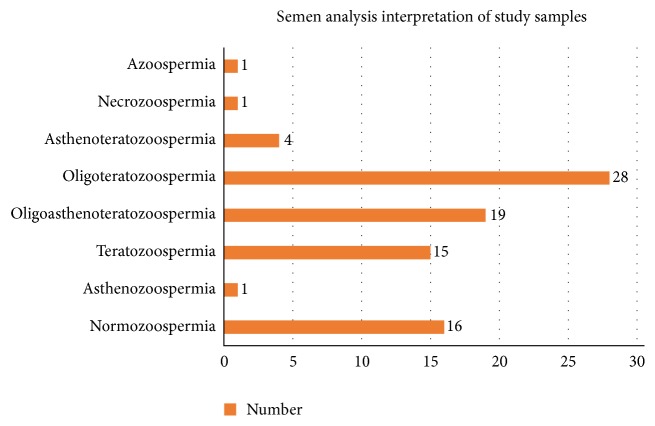
Interpretation of seminal parameters among study population.

Multiple abnormalities such as oligoasthenoteratozoospermia, oligoteratozoospermia, and asthenoteratozoospermia were detected in 19 (22.35%), 28 (32.94%), 4 (4.71%), and 1 (1.17%) specimens, respectively (Figure 2). All the other parameters such as pH, viscosity, volume, liquefaction, and antisperm antibodies were found to be normal.

Asthenozoospermia and teratozoospermia were more common among oligospermic individuals than the individuals with normal sperm count and the association was found to be statistically significant (Table 1).

Bacteriospermia was seen in 30 (35.29%) samples.* Enterococcus faecalis* (30%) was the most common organism isolated followed by Coagulase negative* Staphylococcus* (23.33%),* Staphylococcus aureus* (20%), and* E. coli* (10%). Other less frequently isolated organisms were* Klebsiella pneumoniae* (6.66%),* Proteus* sp. (6.66%), and* Citrobacter* sp. (3.33%) (Table 2). One of the* K. pneumoniae* was isolated from a case where necrozoospermia was demonstrated. Also noted were leukospermia, altered color, and odor of the semen in this patient.

No definite relationship was established between semen parameters and bacteriospermia (Table 3).

The altered semen quality among different bacterial species also lacks significant association (Table 4).

Antibiotic susceptibility testing using Kirby-Bauer disc method showed one vancomycin resistant* E. faecalis* and three carbapenem resistant Gram-negative isolates (Table 5).

## 5. Discussion

The incidence of infertility increases as the age of both partners increases [13]. Due to varied reasons, marriages and subsequently the age of first child birth are getting delayed, thereby subsequently increasing the infertility rates. Our study also depicts the same with majority of men (78.82%) aged above 30 years undergoing infertility evaluation. Based on the sperm count, nearly half of the study population was found to be oligospermic, as normal sperm count was observed only in 55.3% of study population.

When analysing each semen parameter individually, teratozoospermia (81.17%) was the most common abnormality observed in this study. Our findings go hand in hand with those of Jajoo and Kalyani [14] and Owolabi et al. [15] stating teratozoospermia as the most common abnormality in infertile men.

Combining all parameters, abnormal semen quality was observed in 81.17% of samples and oligoteratozoospermia was the most common form of seminal presentation in this study while oligozoospermia was the commonest form of seminal male infertility in a study by Alekwe et al. [16].

Abnormalities in motility and morphology were more commonly observed among oligospermic individuals than normospermic and this was statistically significant. Owolabi et al. also reported the same [15].

The prevalence of bacteriospermia in this study was 35.3%. Similar prevalence rate (35.22%) was observed in a study by Golshani et al. [17]. Higher prevalence rates of 42.9%, 51.7%, 52.5%, 65.7%, 66%, and 79% were shown by Mogra et al. [18], Cotell et al. [19], Alekwe et al. [16], Isaiah et al. [20], Merino et al. [21], and Damirayakhian et al. [22], while Domes et al. have demonstrated lesser (15%) prevalence rate of bacteriospermia [11].

Among the various bacteria isolated,* E. faecalis* (30%) predominated in this study which is in comparison with other studies by Domes et al. [11], Balmelli et al. [23], Ostwal [24], and Moretti et al. [25]. However, in few studies,* Staphylococcus aureus* and* S. epidermidis* were found to be more commonly isolated [15, 16, 20, 26–28].

None of the semen parameters were significantly affected in this study's bacteriospermic samples, which have been reported in many other studies investigating bacteriospermia in infertile men [11, 19, 29, 30]. Though we come across a good number of works on asymptomatic bacteriospermia, query on its impact on infertility still exists. Cotell et al. and few others have shown the presence of bacteria in semen as just contaminants [19]. Ombelet et al. described that the presence of bacteria in seminal fluid in infertile men could be similar to fertile, with its clinical significance being still a matter of debate [31]. However, bacteriospermia was significantly associated with semen quality in studies by Isaiah et al. [20] (with sperm count) and Golshani et al. (with motility and morphology) [17].

Concerning* E. faecalis, Staphylococcus aureus*, and Coagulase negative* Staphylococci*, despite being more frequently isolated from teratozoospermic men, the finding was not supported by statistical significance in our work. This might be due to the lesser number of isolates observed in this study. A work done by Alekwe et al. projected that* Enterococcus faecalis* were commonly isolated from teratozoospermic infertile males with a statistically significant association (*P* < 0.001) [16]. Also, Moretti et al. studying the ultrastructural effect of* E. faecalis* on spermatozoa concluded that it compromises as the sperm concentration and morphology but not sperm motility [25].

We had an isolate of* Klebsiella* in a case where semen parameters were negatively influenced by this bacterium. Though we lack statistical significance, we can probably say that* Klebsiella* could be the cause of necrozoospermia in this individual as the semen had plenty of pus cells and yellowish colour with offensive odor. No definite relation was obtained between the type of bacteria and semen parameters in this study which is also shown by Mogra et al. [18]. However, association of* E. coli* with reduced semen density and motility has been observed in few studies [32–35]. Similarly negative impact of* E. faecalis, E. coli*,* U. urealyticum*, and* M. morganii* on sperm concentration and morphology was shown by Moretti et al. [25]. Rodin et al. demonstrated that samples with* Streptococcus viridans* and* E. faecalis* negatively affected semen quality [26]. Oyeyipo et al. observed that* S. aureus, E. coli,* and* S. saprophyticus* caused the most negative effect on sperm morphology and motility [36].

Merino et al. [21] and Ekhaise and Richard [27] showed that motile spermatozoa and viability were lower when the microorganisms were present in the semen. Huwe et al. also observed the same finding in* Candida albicans* along with the other bacteria [37]. Bornman established that the morphological abnormalities of spermatozoa were seen in samples where* Mycoplasma* and* Ureaplasma* had grown [38]. Eggert-Kruse et al. [39] and Kiessling et al. [40] have demonstrated that anaerobes do grow in semen in significant proportion; however, their role in infertility yet remains to be studied.

Despite works being done using a standard definition for bacteriospermia, it is still a dilemma to call it mere contaminants or asymptomatic infection. Such an inability to have a clear cut differentiation may account for not demonstrating any deterioration in the semen parameters in bacteriospermic samples in this study. Added to this could be the absence of methods employed to detect other bacteria like* Chlamydia*,* Mycoplasma*,* Ureaplasma*, and anaerobes in this study due to technical difficulties. All the above findings question the routine treatment of asymptomatic bacteriospermic men with antibiotics. In this study, we had one vancomycin resistant* Enterococcus faecalis* and three carbapenem resistant Gram-negative bacilli.

To conclude, we had not obtained any positive association of asymptomatic bacteriospermia and infertility in this study. However, as the semen analysis is quite different from other investigations in being having the potential to determine the future generation, further precise studies should be planned to highlight the role of asymptomatic bacteriospermia in infertility by conducting randomized control trials and evaluating the treatment outcomes of asymptomatic bacteriospermia.

## Figures and Tables

**Table 1:** Comparison of abnormal semen parameters between individuals with normal sperm count and oligozoospermia.

Parameter	Oligozoospermia	Normal sperm count	*P* value
Motility (<32%)	16	8	0.0208
Morphology (<4%)	38	31	0.0002

**Table 2:** Various organisms isolated and their frequency.

Organism	Number	Percentage
*Enterococcus faecalis*	9	30
Coagulase negative *Staphylococcus*	7	23.33
*Staphylococcus aureus*	6	20
*Escherichia coli*	3	10
*Klebsiella pneumoniae*	2	6.66
*Proteus *sp.	2	6.66
*Citrobacter* sp.	1	3.33

**Table 3:** Comparison of semen parameters among bacteriospermic and nonbacteriospermic samples.

Parameter	Bacteriospermia	Nonbacteriospermia	*P* value
Sperm concentration			0.1838
<15 million/mL	10	28
≥15 million/mL	20	27

Motility			0.8124
<32%	8	16
≥32%	22	39

Morphology			0.2820
<4%	22	47
≥4%	8	8

**Table 4:** Semen parameters among various bacterial species.

Bacteria	Count		Motility		Morphology	
	≥15 million/mL	<15 million/mL	≥32%	<32%	≥4%	<4%
*E. faecalis*	5	4	6	3	2	7
CoNS	4	3	6	1	1	6
*S. aureus*	4	2	4	2	1	5
*E. coli*	3	0	3	0	1	2
*K. pneumoniae*	1	1	1	1	1	1
*Proteus *sp.	2	0	1	1	2	0
*Citrobacter *	1	0	1	0	0	1

*P* values were >0.05.

**Table 5:** Antibiotic susceptibility profile of bacterial isolates.

Organism	Cx	Va	A	G	Ak	Cf	Caz	Imp
*E. faecalis*		8 (89)	6 (67)	6 (67)		8 (89)		
*S. aureus*	6 (100)	6 (100)	0 (0)	3 (50)		4 (67)		
*Staphylococcus *sp.	5 (71.4)	7 (100)	2 (28.6)	7 (100)		7 (100)		
*E. coli*	—	—	2 (66.7)	2 (66.7)	3 (100)	2 (66.7)	2 (66.7)	3 (100)
*K. pneumoniae*	—	—	0 (0)	2 (100)	2 (100)	2 (100)	1 (50)	1 (50)
*Proteus* sp.			1 (50)	2 (100)	2 (100)	2 (100)	1 (50)	2 (100)
*Citrobacter* sp.			1 (100)	0 (0)	1 (100)	0 (0)	1 (100)	1 (100)

Cx, cefoxitin; Va, vancomycin; A, ampicillin; G, gentamycin; Ak, amikacin; Cf, ciprofloxacin; Caz, ceftazidime; Imp, imipenem.
